# Localization of MLH3 at the Centrosomes

**DOI:** 10.3390/ijms150813932

**Published:** 2014-08-11

**Authors:** Lennart M. Roesner, Christian Mielke, Silke Faehnrich, Yvonne Merkhoffer, Kurt E. J. Dittmar, Hans G. Drexler, Wilhelm G. Dirks

**Affiliations:** 1 Leibniz-Institute DSMZ-German Collection of Microorganisms and Cell Cultures, Department of Human and Animal Cell Lines, Braunschweig 38124, Germany; E-Mails: sif@dsmz.de (S.F.); yvonne.merkhoffer@dsmz.de (Y.M.); hdr@dsmz.de (H.G.D.); wdi@dsmz.de (W.G.D.); 2 Hannover Medical School, Division of Immunodermatology and Allergy Research, Department of Dermatology and Allergy, Hannover 30625, Germany; 3 Institute of Clinical Chemistry and Laboratory Diagnostics, Heinrich-Heine-University, Medical School, Düsseldorf 40225, Germany; E-Mail: christian.mielke@med.uni-duesseldorf.de; 4 Helmholtz Centre of Infection Research, Braunschweig 38124, Germany; E-Mail: ked@helmholtz-hzi.de

**Keywords:** DNA mismatch repair (MMR), *MLH3*, centrosome

## Abstract

Mutations in human DNA mismatch repair (MMR) genes are commonly associated with hereditary nonpolyposis colorectal cancer (HNPCC). MLH1 protein heterodimerizes with PMS2, PMS1, and MLH3 to form MutLα, MutLβ, and MutLγ, respectively. We reported recently stable expression of GFP-linked *MLH3* in human cell lines. Monitoring these cell lines during the cell cycle using live cell imaging combined with confocal microscopy, we detected accumulation of MLH3 at the centrosomes. Fluorescence recovery after photobleaching (FRAP) revealed high mobility and fast exchange rates at the centrosomes as it has been reported for other DNA repair proteins. MLH3 may have a role in combination with other repair proteins in the control of centrosome numbers.

## 1. Introduction

Mutations in human DNA mismatch repair (MMR) genes are commonly associated with hereditary nonpolyposis colorectal cancer (Lynch syndrome). To form the MMR protein complexes, MSH2 heterodimerizes with MSH3 and MSH6, while MLH1 heterodimerizes with PMS2, PMS1, and MLH3 to form MutSα, MutSβ, MutLα, MutLβ, and MutLγ, respectively. While MutLβ is the key player in MMR, reports on the function of MutLγ concentrate on observations in meiotic recombination events. Knockout-mice revealed a crucial role of MutLγ in crossing-over during meiosis [1,2,3,4], but investigating human MutLγ was hampered by a lack of appropriate and accessible experimental systems. Endogenous *MLH3* expression is hardly detectable in human cells since being expressed up to 60 times lower than other MMR proteins [5] and stable recombinant expression could be shown to interfere with cell physiology and is reported to be toxic to human cell lines [5,6]. Recently, we reported for the first time stable expression of recombinant green fluorescent protein (GFP)-linked *MLH3* in human cell line [7]. Using multicistronic vector systems, gene expression was observed to be tolerated if quenched to a low level. Biological function of recombinant MLH3 was confirmed by exclusive nuclear localization, the ability to bind endogenous partner proteins and a fast recruitment to sites of UV laser-induced DNA damage. To investigate the behaviour of MLH3 in the cell, the stable transfected cell lines were monitored during the cell cycle using live cell imaging combined with confocal microscopy.

## 2. Results and Discussion

**Figure 1:**
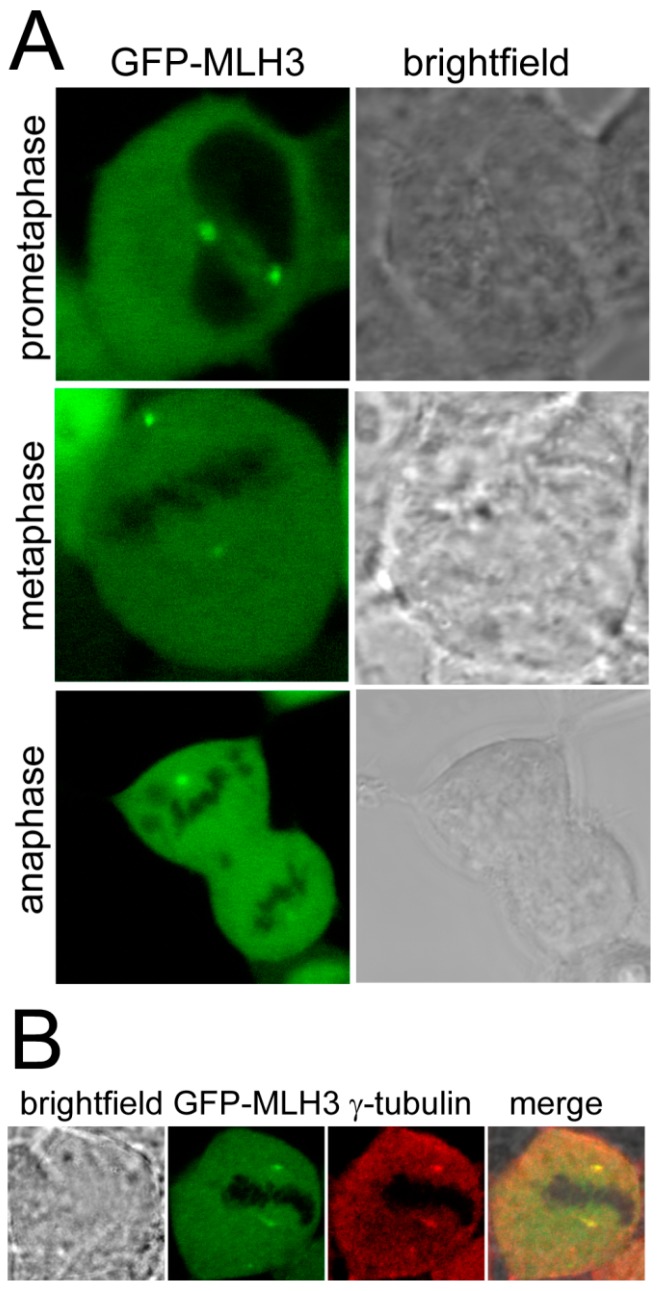
MLH3 accumulates at the centrosomes. (**A**) Confocal microscopy of stably transfected cells (cell line 293), in different stages of mitosis as indicated. GFP-MLH3 accumulates at two foci per cell. Exemplary pictures were chosen, where both fluorescent foci were detected in the same focal pane; and (**B**) Immunostaining of formalin-fixed and permeabilized stably transfected cells using anti-γ-tubulin antibody and a secondary antibody coupled to tetramethylrhodamine.

We detected accumulation of GFP-MLH3 forming two distinct fluorescent foci per cell. The position of the two foci on each side of the aequatorial plane during the different phases of the cell cycle suggested that these might resemble accumulation of MLH3 at the centrosomes (Figure 1a). Immunostaining using an antibody against the centrosome-associated γ-Tubulin (Sigma–Aldrich, St. Louis, MO, USA) confirmed this hypothesis by co-localization with GFP-MLH3-foci (Figure 1b).

**Figure 2:**
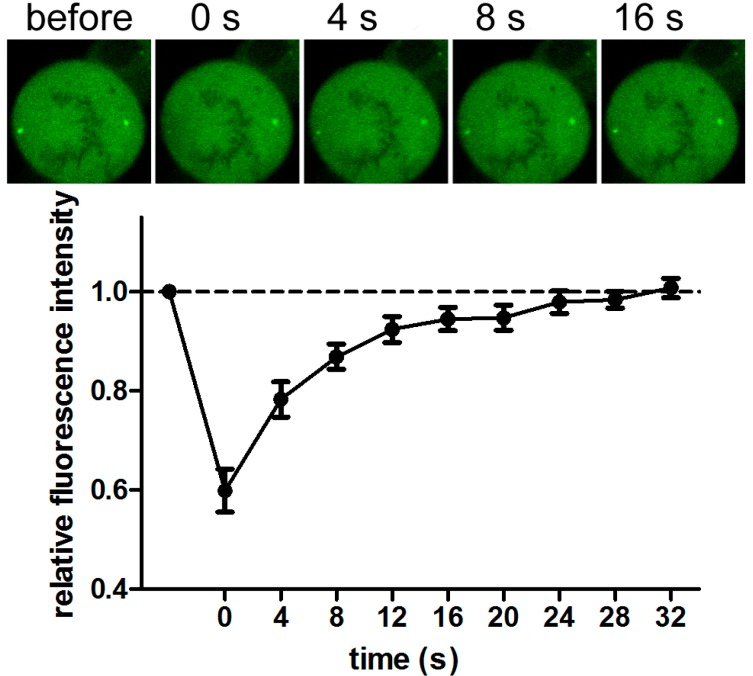
Fluorescence recovery after photobleaching (FRAP) of centrosomal GFP-MLH3 foci. Data shown represents eight independent measurements. Error bars represent the standard error of the mean.

To investigate the nature of these accumulation foci, fluorescence recovery after photobleaching (FRAP) technique was applied. After photobleaching of one of two foci (compare “before” to “0 s”-picture), every 4 s an image was taken. Recovery of signal relative to the other centrosomal foci is achieved within 28 s. Fluorescence recovery to 100% was observed in eight independent measurements (Figure 2). This high mobility resembles fast exchange rates at the centrosomes, which has been reported also for other DNA repair proteins [8,9].

By taking advantage of the capacity of confocal microscopy to eliminate any reflected light from out of the focus, we were able to detect structures within the cell, that are invisible in epifluorescence microscopes. Of course, recombinant expression always harbours the danger of producing artefacts by vast over-expression. In our hands, we achieved a remarkably low expression level applying a multicistronic expression system [7], which turned out to bypass the described toxicity of overexpressed MLH3 [5,6]. Furthermore, the centrosome-associated localization could only be observed for GFP-MLH3, not GFP-MLH1, GFP-PMS1, or GFP-PMS2, where even higher expression levels are achieved by exogenous expression (data not shown). Without the possibility to stain endogenous MLH3 due to the lack of suitable antibodies, we therefore regard our expression system as the best model currently available to study the cellular localization of MLH3.

However, a potential function of MLH3 at the centrosomes may lie in the control of centrosome numbers. If not under proper control, a multipolar spindle apparatus, due to supernumerary centrosomes, leads to a defective mitosis and is described for a number of different cancer types [10]. Regulation of centrosome number is most probably achieved by several spindle assembly checkpoints [11], where repair proteins can trigger a cell cycle arrest in case of errors. Up to now, a number of repair proteins of the homologous recombination and non-homologous end joining-pathways have been found to be involved [12]. However, also for the MMR, key player MSH2 functions in centrosome maintenance are reported [13]; *Msh2*^−/−^ mice showed an increased number of centrosomes in every third cell as well as modifications at the telomers. The localization of MSH2 at the centrosomes was reported subsequently by Narine *et al.*, 2010 [14]. Furthermore, MMR family member MSH4 may be associated to centrosomes, as binding to the microtubule-synthesis associated protein VBP1 (von Hippel–Lindau binding protein 1) has been shown [15,16]. Since MLH3 harbours a binding site to MSH4 [4], this might be the link attracting the protein to the centrosomes during mitosis and an interesting target for further investigation.

## 3. Experimental Section

### 3.1. Cell Lines

Human embryonal kidney cell line 293 (DSMZ, Braunschweig, Germany, ACC 305) was grown in 90% Dulbecco’s MEM supplemented with 10% fetal bovine serum, 100 U/mL penicillin, 100 μg/mL streptomycin and Glutamax-I (Invitrogen, Karlsruhe, Germany) at 37 °C with 10% CO_2_. Subculturing was achieved by splitting confluent cultures about 1:5 every 3 days by detaching cells by tapping the culture flasks. Stable expressing cell lines were generated as described before [7]. Briefly, *MLH3* was amplified by RT-PCR from primary human MRC-5 lung cells. cDNAs were fused *N*-terminally and in frame to GFP and *C*-terminally to an internal ribosomal entry site (IRES), while latter one was fused to the selectable marker gene puromycin-*N*-acetyltransferase to be expressed in a bi- or tricistronic manner. Cells were transfected with plasmid DNA using SuperFect Transfection Reagent according the manufacturer’s guideline (Qiagen, Hilden, Germany). Forty-eight hours after transfection, selective pressure was applied by 0.7 μg/mL puromycin. After 14 to 20 days clones were isolated, expanded and analysed for fluorescence and exclusive nuclear localization.

### 3.2. Immunofluorescence Staining

Cells were grown in chamberslides (Thermo Scientic Nunc LabTek, Waltham, MA, USA) upon semi-conluency and were fixed subsequently using 4% formaldehyde/PBS for 15 min at 37 °C. After permeabilization over night in 0.1 M Tris–HCl/50 mM EDTA/0.5% TritonX-100 blocking was performed for 2 h with 3% bovine serum albumin/0.1% Tween20. Antibodies were applied in PBS/1% fetal bovine serum/0.1% Tween20 for 2 h each. Mouse monoclonal anti-γ-tubulin antibody (Sigma–Aldrich, St. Louis, MO, USA) was applied 1:50, followed by goat-anti-mouse-tetramethylrhodamine (Southern Biotechnology, Birmingham, AL, USA) 1:200.

### 3.3. Microscopy

Microscopy was performed on a Zeiss LSM 510 Meta (Zeiss, Jena, Germany) using the 488 nm laser line to excite GFP and a Plan-NeoFluar 40×/1.3 oil immersion objective. The pinhole was set to 1–2 mm. Live cell imaging experiments were performed at physiological conditions in chamberslides, using a heating stage in combination with an incubation unit and an active gas mixer (Ibidi, Martinsried, Germany) to achieve 37 °C temperature and physiologic CO_2_-content. Furthermore, an objective heater (Zeiss) excluded any undesired cooling by the objective itself.

### 3.4. Fluorescence Recovery after Photobleaching (FRAP)

FRAP was carried out by bleaching a defined area inside the nucleus with 25% laser power, 100% transition and 20 iterations. Several images were taken before bleaching, followed by one image directly after the bleach and consecutive images every 2 s with 5% transition. Fluorescence intensity was measured at each time point. Background was subtracted before fluorescence loss due to monitor bleaching was normalized, to calculate relative intensity *I*_rel_ = (*T*_0_ × *I*_t_)/(*T*_t_ × *I*_0_), where *T* is the total cellular intensity at the time points 0 and t and *I* the average intensity of the bleached region at the time points 0 and t.

## 4. Conclusions

MMR protein *MLH3* accumulates with fast exchange rates at the centrosomes.
